# Characterization and genomic study of “phiKMV-Like” phage PAXYB1 infecting *Pseudomonas aeruginosa*

**DOI:** 10.1038/s41598-017-13363-7

**Published:** 2017-10-12

**Authors:** Xinyan Yu, Yue Xu, Yu Gu, Yefei Zhu, Xiaoqiu Liu

**Affiliations:** 1 Key Laboratory of Pathogen Biology of Jiangsu Province, Department of Microbiology, Nanjing Medical University, Nanjing, 211166 China; 2 The Second Affiliated Hospital of Nanjing Medical University, Nanjing, China

## Abstract

Bacteriophage PAXYB1 was recently isolated from wastewater samples. This phage was chosen based on its lytic properties against clinical isolates of *Pseudomonas aeruginosa* (*P. aeruginosa*). In the present study, characterized PAXYB1, clarified its morphological and lytic properties, and analyzed its complete genome sequence. Based on the morphology of PAXYB1, it is a *Podoviridae*. The linear GC-rich (62.29%) double-stranded DNA genome of PAXYB1 is 43,337 bp including direct terminal repeats (DTRs) of 468 bp. It contains 60 open reading frames (ORFs) that are all encoded within the same strand. We also showed that PAXYB1 is a virulent phage and a new member of the phiKMV-like phages genus. Twenty-eight out of sixty predicted gene products (gps) showed significant homology to proteins of known function, which were confirmed by analyzing the structural proteome. Altogether, our work identified a novel lytic bacteriophage that lyses *P. aeruginosa* PAO1 and efficiently infects and kills several clinical isolates of *P. aeruginosa*. This phage has potential for development as a biological disinfectant to control *P. aeruginosa* infections.

## Introduction

*Pseudomonas aeruginosa (P. aeruginosa*) is an opportunistic human pathogen that is widely distributed in various environments. *P. aeruginosa* can cause chronic infections or acute life-threatening infections in patients hospitalized with cystic fibrosis, otitis media, keratitis, and severe burns and in immunocompromised individuals^1,2^. *P. aeruginosa* is a highly adaptable bacterium, making it one of the most resistant microorganisms in the presence of long-term antibiotic therapy. The continuing increase in the antibiotic resistance of clinical *P. aeruginosa* strains worldwide and a shortage of new drugs being discovered in the pipeline have led to an urgent need to explore more alternative strategies such as phage therapy, which has been demonstrated as a promising way to control *P. aeruginosa*-mediated infections^3^.

Several studies have shown the potential of phage application into the treatment of infectious diseases caused by *P. aeruginosa*, particularly in infections caused by multidrug resistant strains^4–6^. Phage therapy has been used for the control and treatment of multidrug-resistant *P. aeruginosa* in lung infections in mouse models and in cystic fibrosis airway lung cells *in vitro* ^7,8^. *P. aeruginosa* phages also have therapeutic effects on infectious keratitis, burn wound infection, gut-derived sepsis and chronic otitis^9–13^. Interestingly, a mixture of six lytic phages was shown to be effective in treating clinical ear infections caused by *P. aeruginosa* ^14^. It is generally accepted that the use of mixtures (cocktails) of phages targeting different hosts counteracts the downsides of phage therapy, such as their relatively narrow host range and emerging bacterial resistance^15^.

Bacteriophages are estimated to be more numerous globally (10^31^) than bacteria^16^. Presently, 2111 phage genomes have been sequenced (from the NCBI database). However, only some of them have been tested for their capacity to target various clinical strains either *in vitro* or in animal models. Amongst the 2111 phages, 152 are *Pseudomonas* phages (from the NCBI database), and more than twenty phages targeting *P. aeruginosa* have been tested in animal models these past few years^17,18^. Compared to the large number of phages on earth, the available number of isolated phages is still relatively limited. An urgent demand for the isolation of potent, strongly lytic, well-characterized phages from the environment and for use in phage therapy is steadily growing^19^. The isolation of new phages against non-laboratory strains of *P. aeruginosa* and an analysis of their genomes and lytic behaviour will lead to a more comprehensive view of the antimicrobial potential of virulent *P. aeruginosa* phages.

Here, we isolated and characterized a new phage infecting *P. aeruginosa* named PAXYB1. It is a *Podoviridae* from its virion structure, and it has a strong ability to infect several clinical isolates of *P. aeruginosa*. The genome sequence of this phage is over 90% identical to other members of the PhiKMV-like virus genus^18,20,21^. We also analyzed the particle protein content of this phage and detected 17 structural proteins. Based on morphological, genomic, and proteomic analysis, PAXYB1 is a new member of the PhiKMV-like virus genus. In-depth characterization of this new phage will contribute to the promising therapeutic use of this phage.

## Results

### Isolation and morphology of the PAXYB1 bacteriophage

**Figure 1:**
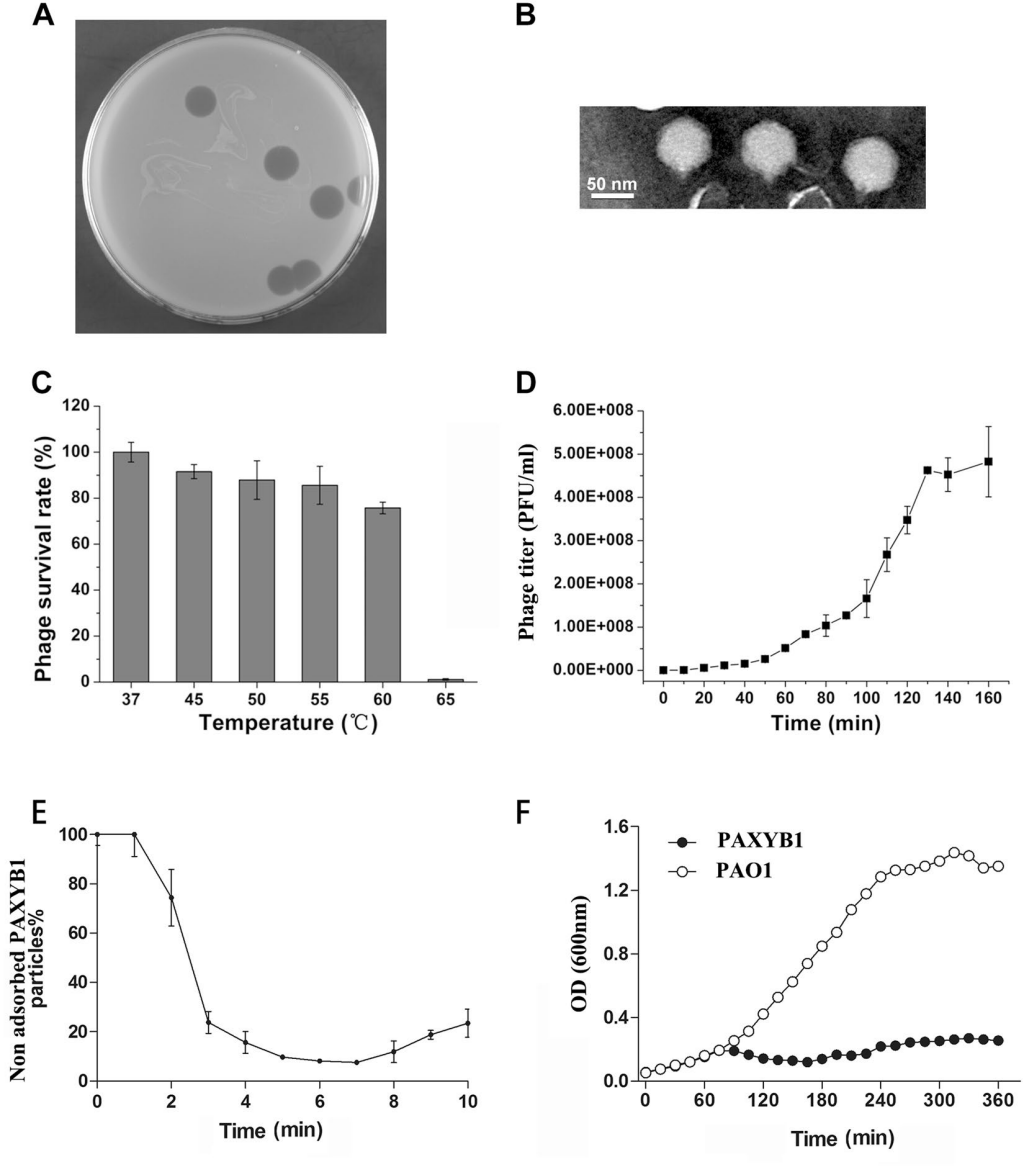
Isolation, morphology and biological properties of phage PAXYB1. (**A**) Plaque morphology of phage PAXYB1. (**B**) Transmission electron micrographs of PAXYB1 phage particles, negatively stained with phosphotungstic acid (2% [wt/vol]). The scale bars represent 50 nm in B. (**C**) Thermo stability of PAXYB1: the phages were incubated at different temperatures for 30 min. (**D**) One-step growth curve of PAXYB1 on *P.aeruginosa* strain PAO1. (**E**) Adsorption assays of PAXYB1 on *P. aeruginosa* strain PAO1. (**F**) Individual *in vitro* lysis kinetics for PAXYB1 at an MOI of 0.001 on the *P. aeruginosa* strain PAO1. Each value is the average from three different cultures ± standard deviation in (**C**–**F**).

The lytic phage PAXYB1 isolated from wastewater was used to infect *P. aeruginosa* strain PAO1. As shown in Fig. 1A, PAXYB1 formed large, clear and round plaques approximately 0.8 cm in diameter after incubation for 12 h at 37 °C. Under the electron microscope, intact PAXYB1 particles had an approximately 50 nm-diameter icosahedral head and a short tail approximately 10 nm in length (Fig. 1B), demonstrating that PAXYB1 belongs to the *Podoviridae* family.

### Host range analysis and thermo stability

To estimate the potential application of PAXYB1 in phage therapy, 20 clinical isolates of *P. aeruginosa* from different sources (Table 1), most of which are multi-drug-resistant, were used to test the lytic spectrum. The results showed that PAXYB1 was able to infect 13 of the 20 tested strains and formed clear plaques on 10 of the strains (Table 1), indicating that PAXYB1 can efficiently infect and kill some clinical isolates of *P. aeruginosa*.

**Table 1:** Test lysis spectrum of phage PAXYB1.

Strains	Resistance	Source	Lysed or not
*P. aeruginosa PAO1*		Standard Laboratory Reference Strain	Clear plaque
*Escherichia coli K*-*12 MG1655*		Standard Laboratory Reference Strain	N
*P. aeruginosa 2320*	Amikacin, minocycline, ticarcillin/clavulanic acid, aztreonam, piperacillin, ceftazidime, ticarcillin	Sputum, Male, 86^#^	N
*P. aeruginosa 2321*	Minocycline, ticarcillin/clavulanic acid, aztreonam, piperacillin, ceftazidime, ticarcillin	Wound secretion, Male, 48^#^	Clear plaque
*P. aeruginosa 2324*	Minocycline, ticarcillin/clavulanic acid, ticarcillin	Wound secretion, Male, 71^#^	Turbid plaque
*P. aeruginosa 2325*	Minocycline	Sputum, Female, 65^#^	Clear plaque
*P. aeruginosa 2357*	Minocycline, ticarcillin/clavulanic acid, ticarcillin	Sputum, Male, 58^#^	N
*P. aeruginosa 2372*	Ampicillin, cefotetan, ceftriaxone, cotrimoxazole, ampicillin/sulbactam, cefazolin	Blood, Female, 77^#^	Clear plaque
*P. aeruginosa 2382*	Amikacin, cefepime, levofloxacin, piperacillin, ceftazidime, ticarcillin, gentamicin, aztreonam, imipenem, meropenem, cefoperazone/sulbactam, ticarcillin/clavulanic acid, piperacillin/tazobactam	Sputum, Male, 87^#^	N
*P. aeruginosa 2383*	Ticarcillin/clavulanic acid, imipenem, aztreonam, ceftazidime, ticarcillin, levofloxacin, cefoperazone/sulbactam	Sputum, Male, 82^#^	Clear plaque
*P. aeruginosa 2384*	Ticarcillin, imipenem, ticarcillin/clavulanic acid	Sputum, Female, 69^#^	Clear plaque
*P. aeruginosa 2387*	meropenem, ticarcillin/clavulanic acid, aztreonam, levofloxacin, ticarcillin, imipenem	Sputum, Male, 69^#^	Clear plaque
*P. aeruginosa 2388*	Amikacin, cefepime, levofloxacin, gentamicin, imipenem, cefoperazone/sulbactam, piperacillin/tazobactam, ampicillin, cefoxitin, ciprofloxacin, tigecycline, cotrimoxazole, amoxicillin/clavulanic acid, cefazolin, ceftriaxone, tobramycin, nitrofurantoin	Urine, Male, 81^#^	Clear plaque
*P. aeruginosa 2395*	Ampicillin, cefotetan, ceftriaxone, cotrimoxazole, ampicillin/sulbactam, cefazolin	Blood, Female, 67^#^	Clear plaque
*P. aeruginosa 2399*	Imipenem	Blood, Female, 73^#^	Clear plaque
*P. aeruginosa 2401*	Amikacin, ticarcillin, gentamicin, imipenem, ticarcillin/clavulanic acid	Sputum, Male, 86^#^	N
*P. aeruginosa 2541*	Amikacin, cefepime, levofloxacin, piperacillin, ceftazidime, ticarcillin, gentamicin, aztreonam, imipenem, meropenem, cefoperazone/sulbactam, ticarcillin/clavulanic acid, piperacillin/tazobactam	Sputum, Male, 50^#^	Clear plaque
*P. aeruginosa 2558*	Ticarcillin, ticarcillin/clavulanic acid	Sputum, Female, 64^#^	Turbid plaque
*P. aeruginosa 2579*	Ticarcillin, imipenem, ticarcillin/clavulanic acid	Urine, Female, 67^#^	N
*P. aeruginosa 2612*	Imipenem	Sputum, Female, 61^#^	N
*P. aeruginosa 2619*	Levofloxacin, piperacillin, ticarcillin, gentamicin, imipenem, meropenem, ticarcillin/clavulanic acid	Sputum, Male, 81^#^	N
*P. aeruginosa 1609443*	Levofloxacin, ticarcillin, gentamicin, imipenem, ticarcillin/clavulanic acid	n.d.	Turbid plaque

Positive results are indicated by “Clear plaque” or “Turbid plaque”, and negative results are indicated by “N”; n.d., no data available; ^#^numbers in the column “source” represent “the age of the individual”.

Assessment of the thermal stability of PAXYB1 revealed that PAXYB1 was active at a wide range of temperatures from 37 °C to 55 °C for 30 min, with more than 70% of the phages still alive even after incubation at 60 °C (Fig. 1C). However, a marked decline of the survival rate was observed at 65 °C, with less than 1% of the phage particles surviving at 65 °C (Fig. 1C). Karumidze’s study showed that the half-lives of *P. aeruginosa* phages vB_Pae-Kakheti25 and vB_Pae-TbilisiM32 are 49 and 46 min at 70 °C, respectively^21^, which indicates that these two phages are more stable than PAXYB1 at higher temperatures. A survey showed that the titres of *P. aeruginosa* phages P2S2 and P5U5 decreased following heating for 10 to 50 min at 45 °C or 60 °C^22^. *Pseudomonas fluorescens* bacteriophage KSL-1 titre reduction was calculated as only 1.1 log at 60 °C^23^. PAXYB1 is relatively stable compared to phages P2S2, P5U5 and KSL-1 at higher temperatures.

### One-step growth curve, adsorption assays and lysis kinetics

To further reveal the lytic cycle of PAXYB1, a one-step growth curve was performed. As shown in Fig. 1D, the latent and rise periods for PAXYB1 were approximately 30 and 100 min, respectively. The burst size was approximately 141 PFU/infected cell.

Adsorption assays showed that PAXYB1 particles efficiently adsorb to *P. aeruginosa* cells (ka = 3.7 × 10^−9^ ml min^−1^), resulting in a 10-fold reduction in nonadsorbed phage particles 7 min after infection (Fig. 1E). This differs compared to LKD16 and phiKMV, both of which failed to produce a clear adsorption curve and showed only minor adsorption to host cells [20 to 30% at an multiplicity of infection (MOI) of 0.001]^24^. However, they are similar to PAK_P3 and LKA1 particles^24,25^, which efficiently adsorb to *P. aeruginosa* cells 4–5 min after infection.

We then determined the lysis kinetics of PAXYB1 on the PAO1 stain at MOI 0.001 in liquid medium (Fig. 1F). The OD values decreased approximately 75 min after incubation with PAXYB1, similar to PAK_P1, PAK_P2, PAK_P4 and PAK_P5, which lyse the PAK-lumi strain after approximately 70 min^26^. PAXYB1 lysis on PAO1 occurred more rapidly compared to LUZ19 (270 min), PhiKZ (210 min) and LBL3 (180 min) on the PAK-lumi stain^26^.

### Analysis and annotation of the PAXYB1 genome

Throughout sequencing of the PAXYB1 genome, reads were assembled into a circular, single contig of 42,869 bp. To verify the obtained nucleotide sequence, the genome DNA was further digested with *Eco*RI (3 sites), *Hin*dIII (5 sites), *Xba*I (2 sites), *Bam*HI (0 site), *Sal*I (2 sites) and *Sac*I (1 site) (Fig. S1). As expected, the genome could not be digested by *Bam*HI and only released a few fragments using the other five restriction endonucleases for digestion. However, the restriction digestions of *Hind*III, *Xba*I, and *Sac*I produced unexpected 1.3, 6.1 and 3.2 kb fragments, respectively, indicating that the PAXYB1 genome is likely linear but not circular. To determine the terminal sequences of the PAXYB1 genome, the 6.1 and 3.2 kb fragments digested by *Xba*I and *Sac*I, respectively, were recovered and directly sequenced with outward pointing primers. As we anticipated, the sequencing reaction came to a stop at the end of the genome. Thus, the PAXYB1 genome is indeed linear and has identical direct terminal repeats (DTRs) of 468 bp.

**Figure 2:**
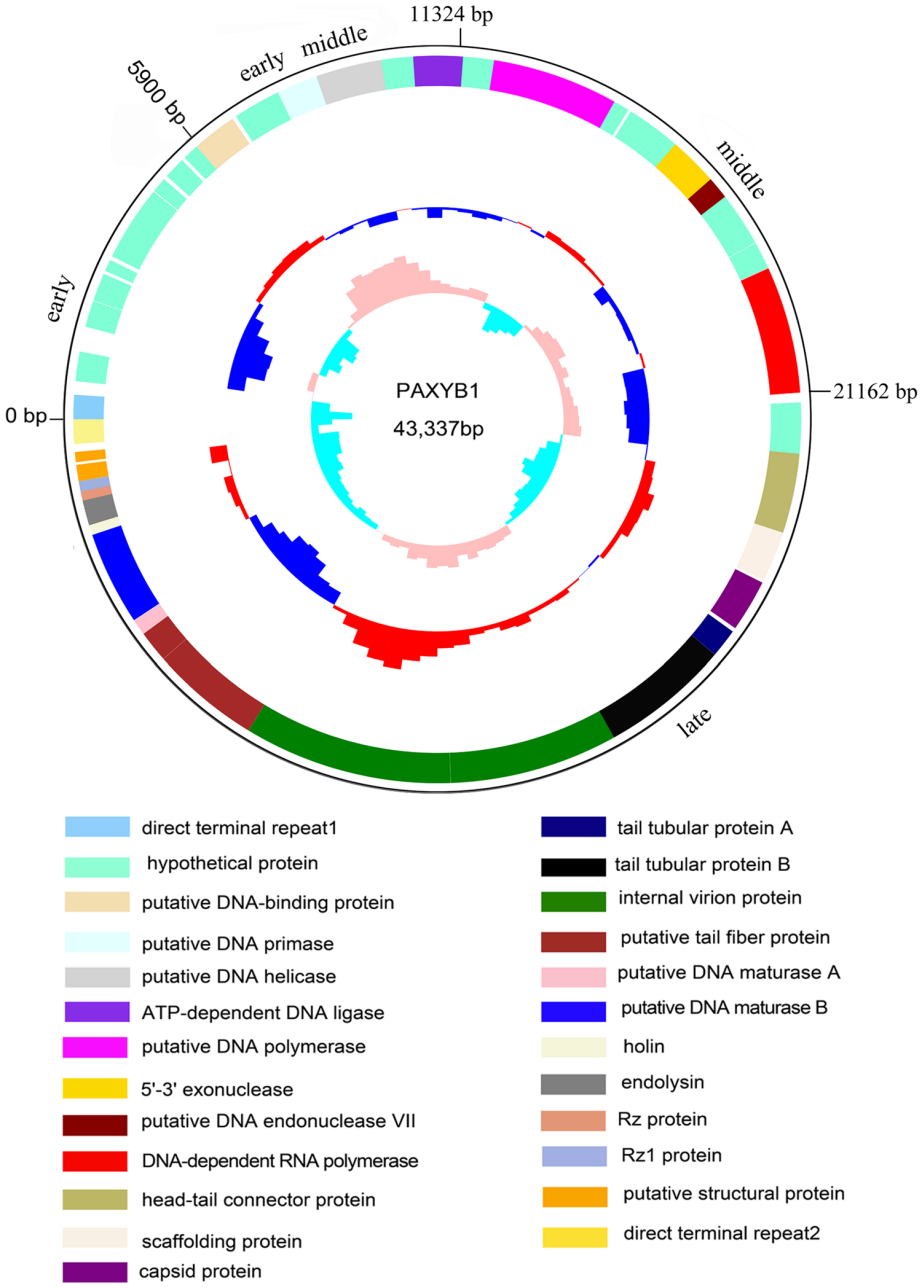
Genome organization of PAXYB1. The first circles represent the ORFs on the sense strands in PAXYB1. Sixty ORFs are marked with different colours according to their different functions and arranged in early, early middle, middle and late clusters. The DTRs are also indicated. The second circle shows the G/C content. Red outward and blue inward indicate that the G/C content of this region is high and less than the average G/C content of the whole genome, respectively. The third circle shows the GC skew.

The reassembled genome of PAXYB1 is 43,337 bp in length and has an average G + C content of 62.29%. Using artemis and blast analysis, 60 ORFs (≥30 aa) were predicted as potential genes, which were encoded within the same direction ((Fig. 2, Table 2). Amongst these, 28 gene products showed significant homology to proteins with known function, and the other 32 genes are presumed to encode hypothetical proteins. No gene related to phage lysogeny was found, confirming the lytic characteristics of phage PAXYB1.

**Table 2:** Predicted ORFs and genes in the PAXYB1 genome.

ORF	Start	Stop	Direction	No. of residues	Mol. mass (kDa)	Calculated isoelectric point	Putative function
ORF1	727	960	+	77	8.3	6.08	hypothetical protein
ORF2	942	1295	+	117	12.8	11.44	hypothetical protein
ORF3	1779	2063	+	94	10.7	10.25	hypothetical protein
ORF4	2063	2290	+	75	8.4	11.00	hypothetical protein
ORF5	2301	2840	+	179	19.6	9.28	hypothetical protein
ORF6	2903	3007	+	34	3.9	9.82	hypothetical protein
ORF7	3010	3129	+	39	4.7	4.50	hypothetical protein
ORF8	3208	3576	+	122	13.1	6.89	hypothetical protein
ORF9	3563	3790	+	75	8.7	10.26	hypothetical protein
ORF10	3787	3972	+	61	7.3	12.43	hypothetical protein
ORF11	3969	4148	+	59	6.6	5.45	hypothetical protein
ORF12	4148	4435	+	95	10.3	6.55	hypothetical protein
ORF13	4432	4668	+	78	8.3	8.29	hypothetical protein
ORF14	4684	4977	+	97	10.8	9.50	hypothetical protein
ORF15	5056	5472	+	138	14.4	5.13	hypothetical protein
ORF16	5541	5900	+	119	13.3	7.89	hypothetical protein
ORF17	5849	6712	+	287	31.3	9.63	putative DNA-binding protein
ORF18	6791	7210	+	139	15.7	4.98	hypothetical protein
ORF19	6981	7520	+	179	20.0	5.88	hypothetical protein
ORF20	7513	7638	+	41	4.2	5.75	hypothetical protein
ORF21	7530	7733	+	67	7.8	9.76	hypothetical protein
ORF22	7706	8530	+	274	31.0	8.91	putative DNA primase
ORF23	8499	9767	+	422	47.4	5.38	putative DNA helicase
ORF24	9757	10377	+	206	22.3	5.21	hypothetical protein
ORF25	10377	11324	+	315	35.1	8.98	ATP-dependent DNA ligase
ORF26	11321	11605	+	94	11.1	11.12	hypothetical protein
ORF27	11602	11922	+	106	12.4	7.83	hypothetical protein
ORF28	11919	14342	+	807	91.8	5.65	putative DNA polymerase
ORF29	14339	14650	+	103	11.3	9.85	hypothetical protein
ORF30	14705	15754	+	349	36.8	5.42	hypothetical protein
ORF31	15754	16695	+	313	35.3	5.87	5′-3′ exonuclease
ORF32	16685	17125	+	146	16.4	9.67	putative DNA endonuclease VII
ORF33	17122	18168	+	348	40.4	9.17	hypothetical protein
ORF34	18178	18552	+	124	14.2	6.59	hypothetical protein
ORF35	18542	18706	+	54	6.3	5.08	hypothetical protein
ORF36	18715	21162	+	815	91.8	6.72	DNA-dependent RNA polymerase
ORF37	21350	21601	+	83	9.4	10.71	hypothetical protein
ORF38	21601	22074	+	157	18.1	6.65	hypothetical protein
ORF39	22019	22315	+	98	10.5	9.77	hypothetical protein
ORF40	22236	22466	+	76	8.5	11.03	hypothetical protein
ORF41	22327	23859	+	510	56.2	5.00	head-tail connector protein
ORF42	23863	24831	+	322	33.2	5.37	scaffolding protein
ORF43	24884	25891	+	335	37.7	5.66	capsid protein
ORF44	25969	26523	+	184	21.2	6.92	tail tubular protein A
ORF45	26526	29006	+	826	92.0	5.99	tail tubular protein B
ORF46	29006	29551	+	181	18.8	8.86	Internalvirion protein
ORF47	29551	32247	+	898	98.2	5.16	Internalvirion protein
ORF48	32251	36264	+	1337	144.0	6.23	Internalvirion protein
ORF49	36266	37021	+	251	28.4	4.56	putative tail fiber protein
ORF50	37021	37479	+	152	16.8	8.00	putative tail fiber protein
ORF51	37472	38377	+	301	33.2	7.71	putative tail fiber protein
ORF52	38381	38986	+	201	22.6	5.65	putative tail fiber protein
ORF53	38986	39291	+	101	11.6	4.76	putative DNA maturase A
ORF54	39300	41105	+	601	67.9	7.21	putative DNA maturase B
ORF55	41102	41302	+	66	7.0	8.93	Holin
ORF56	41299	41781	+	160	17.4	8.77	Endolysin
ORF57	41739	42068	+	109	12.0	9.87	Rz protein
ORF58	41956	42156	+	66	6.9	4.75	Rz1 protein
ORF59	42158	42472	+	104	10.5	5.65	putative structural protein
ORF60	42522	42716	+	64	6.9	5.22	putative structural protein

The overall gene organization of phage PAXYB1 resembles that of the well-known *P. aeruginosa* phage phiKMV^24^. The overall nucleotide sequence of the PAXYB1 genome is approximately 94% identical to that of phiKMV (Table S1). Similar to phage phiKMV^27^, the PAXYB1 genome also comprises early, early middle, middle and late clusters according to the genes’ functions (Fig. 2). The early region (ORF1-ORF16) contains all unknown function genes. The early middle region contains genes that are usually associated with DNA metabolism, including the putative DNA-binding protein, DNA primase, DNA helicase and DNA ligase (Fig. 2). The middle cluster (ORF26-ORF36) is likely responsible for DNA metabolism with DNA polymerase, exonuclease, endonuclease and RNA polymerase. Amongst these genes, the putative DNA-dependent RNA polymerase (ORF36) is located at the end of the DNA metabolism gene cluster, which is the feature of the phiKMV-like phages that is distinguished from other members of the T7 supergroup^27^. The remaining ORFs (ORF37-ORF60), which encode structural and lysis proteins, constitute the late cluster. Based on the similarity of the sequence and gene organization between PAXYB1 and phiKMV^24,27^, we conclude that PAXYB1 is a phiKMV-like virus.

**Figure 3:**
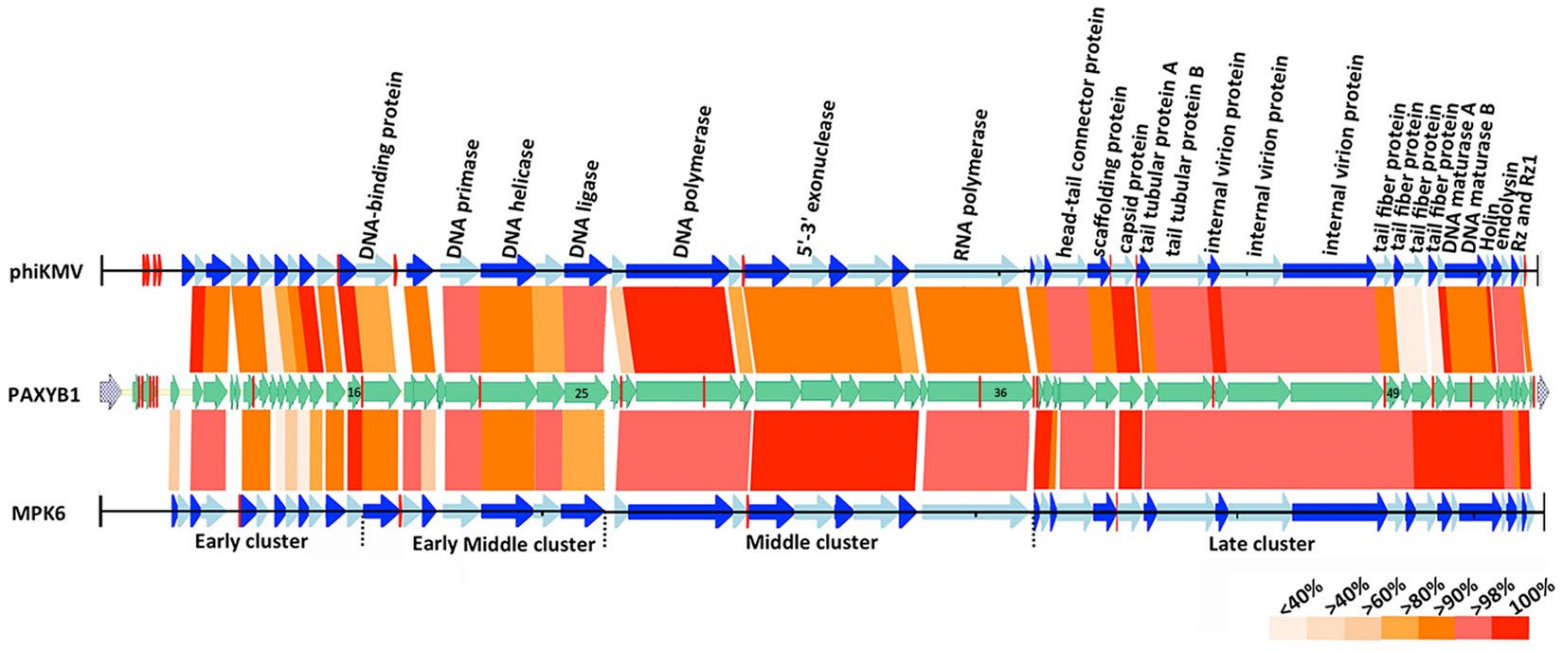
Schematic genomic alignment of the phage PAXYB1 with other two homologous phages. Early, early middle, middle and late clusters of these phages were separated by the dotted lines at the bottom. Homologous ORFs or genes are present, and the percentages of amino acid identities are shown with different colours. Predicted promoters are shown as red bars on the PAXYB1, phiKMV and MPK6 genomes.

We also searched the genome regions of PAXYB1 for promoters. Five putative host promoters (two upstream ORF2 and three upstream ORF3) were identified within the first 1,309 bp of the PAXYB1 genome (Fig. 3), similar to that in the LKD16 genome^24^. PAXYB1 differs from phiKMV, however, which has four potential host sigma70 promoters upstream ORF1^24^. PAXYB1 has a promoter in front of ORF17 (DNA-binding protein) that supports DNA-binding proteins and downstream genes that are located in the early middle cluster (Fig. 3). Moreover, two promoters were predicted in front of ORF37 (the gene after RNA polymerase), which is a starting site of a late cluster (Fig. 3). Eleven promoters were only found in PAXYB1 but not in the other two phiKMV phages (Fig. 3), suggesting slight differences in transcriptional regulation amongst phiKMV-like phages.

### Comparison of the genomes of PAXYB1 and other phiKMV-like phages

To investigate the relationship between newly isolated PAXYB1 and other phiKMV-like phages, we performed a blastn analysis of the entire PAXYB1 genome against the nucleotide collection database at NCBI. As expected, the PAXYB1 genome is highly homologous (coverage 86–97%, identity 91–99%) to 15 other phiKMV-like phages. Their genomic properties are summarized in Table S1. We observed that although isolated from different sources and areas all over the world, many characteristics of the 16 phages are remarkably similar, including the genome size and G + C content. Ten of these sixteen phages were determined to have a similar size (413–488 bp) of DTRs (Table S1).

Most of the genomic diversity of these 16 phages is displayed within the early/early middle clusters, as frequently observed in phage genomes (Fig. S2). A second hot spot of diversity is found within the cluster encoding the tail fibers (Fig. S2). To further investigate the differences between tail fiber proteins of PAXYB1 and other phiKMV-like phages, we performed a protein BLAST analysis to search the homologues of the four proteins in other phages. The global identities between the homologous proteins were then determined by the EMBOSS Needle tool at EMBL-EBI. As shown in Table S2, in other phiKMV-like phages, the homologues of four tail fiber proteins are also arranged sequentially. Gp49 of PAXYB1 is considerably conserved in phiKMV-like phages (identity 83.7–100%), whereas the other three tail fiber proteins vary significantly (28.1–99.3%, 24.9–100% and 34.3–100% of identity, respectively).

**Figure 4:**
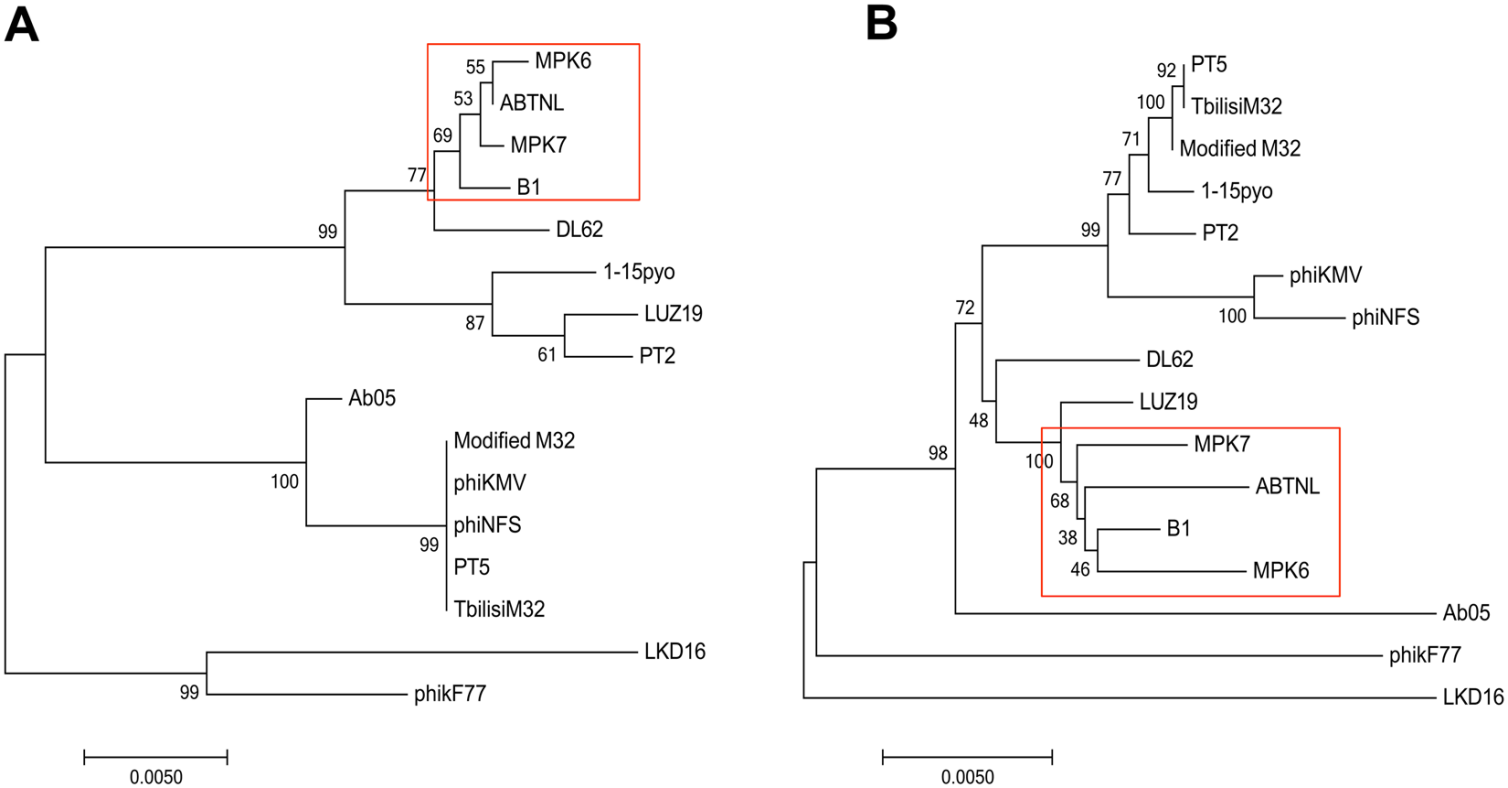
Phylogenetic tree based on RNAP and whole genome sequence comparisons of selected phages. RNAP (**A**) and whole genome sequence comparisons (**B**) were compared using the ClustalW program, and the phylogenetic tree was generated using the neighbour-joining method with 1000 bootstrap replicates. PAXYB1 in brief is “B1” in these phylogenetic trees.

Considering the complexity of the evolutionary relationship of the phiKMV-like phages group, phylogenetic analyses were performed based on RNAP (Fig. 4A) and whole genome sequence comparisons (Fig. 4B). In the two phylogenetic trees, the results showed that 16 phages were both classified into three branches. Even though the two trees report distinct evolutionary relationships between the phages, they both indicate that PAXYB1, MPK6, ABTNL and MPK7 are more closely related between them and that PAXYB1 is distantly related with phiKMV. Combined with the overall nucleotide blast result (Fig. S2), PAXYB1 has the closest relationship with MPK6. In Fig. 3, almost all homologous proteins between PAXYB1 and MPK6 display more than 80% identity, confirming that these two phages are closely related. The most significantly different region between these two phages is gp11-13 (all hypothetical proteins in phiKMV-like phages). There are no homologous proteins of gp11 and gp13 found in MPK6, whereas the gp12 amino acid sequence is 61.9% identical to the corresponding MPK6 protein (Fig. 3). Gp3 and gp19 of PAXYB1, two hypothetical proteins, also exhibited slightly lower identities (64.1% and 50.3%, respectively) with MPK6 (Fig. 3). Gp11-13, gp3, and gp19 have homologues in other phiKMV-like phages, such as MPK7, phiKF77 and phiKMV. These five genes are located in low-identity genomic regions of phiKMV-like phages (Fig. S2), indicating that they are likely highly variable in the process of phage evolution. In agreement with the evolutionary relationship, the PAXYB1 genome is relative different from that of phiKMV. Three out of four putative tail fiber proteins, gp50-52 of PAXYB1, show considerably low homologies with the corresponding proteins of phiKMV (identities of 28.1%, 24.9% and 34.3%, respectively) (Table S2). The difference of tail fiber proteins of these phages might cause difference in their host range.

### Structural proteins of phage PAXYB1

**Figure 5:**
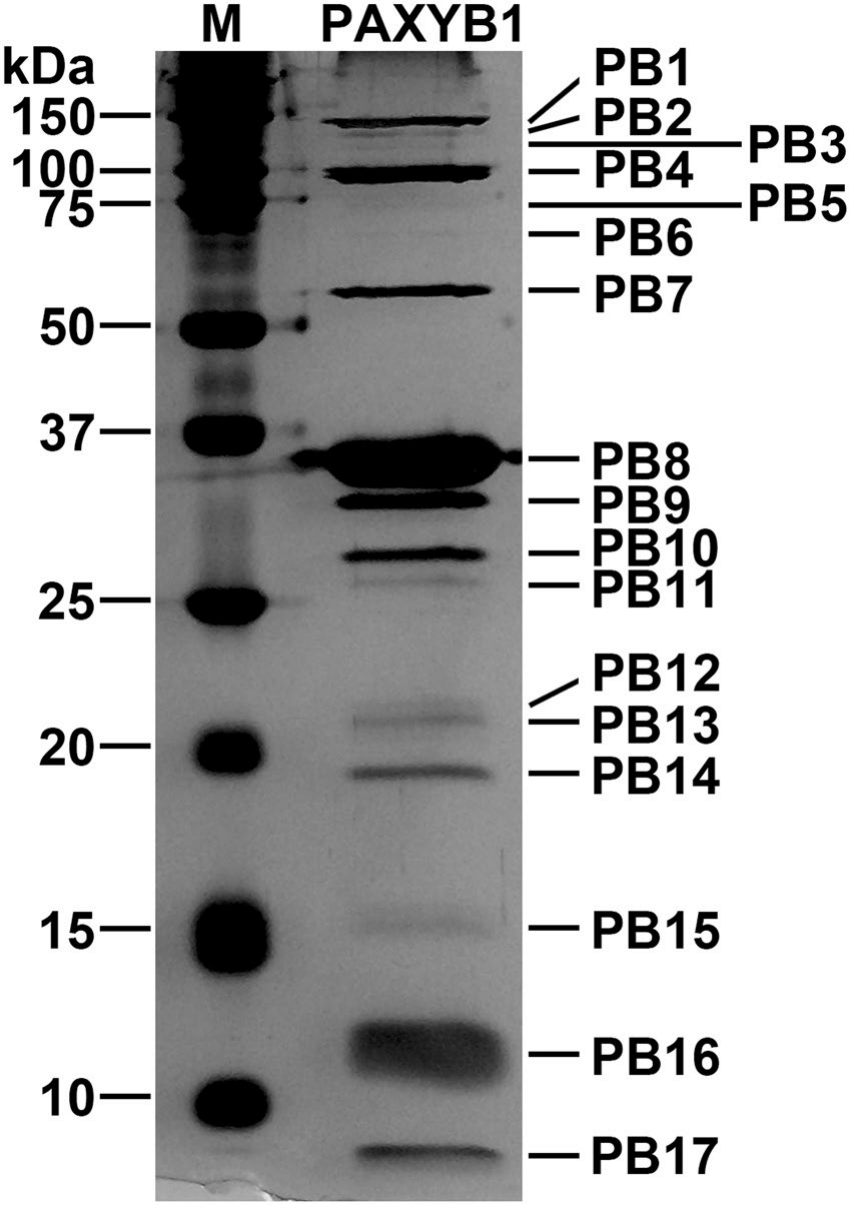
PAXYB1 virion structural proteins. The structural proteins of purified PAXYB1 particles were separated by SDS-PAGE and stained with silver. The positions of the PAXYB1 structural proteins are indicated on the right. Molecular mass markers are shown on the left.

The highly purified PAXYB1 particles were separated on SDS-PAGE and stained with silver (Fig. 5, Fig. S3). At least 17 protein bands with molecular masses of 6.9–144 kDa were observed. In the previous studies, 12, 13 and 18 proteins from phage phiKMV, LKD16 and M32 were detected, respectively^21,24,28^. To identify as many structural proteins as possible, whole phage PAXYB1 particles were directly digested with trypsin and then detected by mass spectrometry. The results showed that 17 structural proteins of PAXYB1 were detected (Table 3). Most of them were also found in the other three PhiKMV-like phages. Scaffolding protein was also confirmed as a structural protein, similar to the phiKMV phage^28^. Furthermore, three additional proteins (gps16, 50, and 57) that were not previously identified were also found in the PAXYB1 virions. Amongst these proteins, gp16, located in the early region, was predicted to be a hypothetical protein in PAXYB1. Another two proteins are located in the late region of the PAXYB1 genome. Gp50 is predicted as one of the tail fiber proteins of PAXYB1. Gp57 (Rz protein) is a member of the lysis cassette that is highly conserved amongst phiKMV-like viruses and organized with four overlapping genes^27^.

**Table 3:** Mass spectrometry data for PAXYB1.

PAXYB1 protein	Predicted function	Mol. mass (kDa)	No. of peptides	No. of unique peptides	Sequence coverage (%)	Protein bands	Previous identification in φKMV	Previous identification in LKD16	Previous identification in M32
ORF16	hypothetical protein	13.3	4	4	37.82	PB16	−	−	−
ORF39	hypothetical protein	10.5	38	7	81.63	PB16	+	+	+
ORF41	head-tail connector protein	56.2	89	26	53.33	PB7	+	+	+
ORF42	scaffolding protein	33.2	3	3	13.04	PB9	+	−	−
ORF43	capsid protein	37.7	670	33	85.07	PB8	+	+	+
ORF44	tail tubular protein A	21.2	12	6	32.61	PB13	+	+	+
ORF45	tail tubular protein B	92.0	82	36	46.97	PB4	+	+	+
ORF46	Internalvirion protein	18.8	21	8	44.20	PB14	−	+	+
ORF47	Internalvirion protein	98.2	147	49	52.00	PB4	+	+	+
ORF48	Internalvirion protein	144.0	159	61	47.57	PB1	+	+	+
ORF49	putative tail fiber protein	28.4	38	13	52.59	PB11	+	+	+
ORF50	putative tail fiber protein	16.8	5	2	21.71	PB15	−	−	−
ORF51	putative tail fiber protein	33.2	40	9	28.90	PB9	+	+	+
ORF52	putative tail fiber protein	22.6	25	9	48.76	PB12	−	+	+
ORF57	Rz protein	12.0	2	2	12.84	PB16	−	−	−
ORF59	putative structural protein	10.5	93	6	83.65	PB16	+	+	+
ORF60	putative structural protein	6.9	8	3	43.75	PB17	+	+	+

The proteins detected by MS are listed with their predicted functions and molecular mass. Molecular mass was calculated from the gene sequence. The number of identified peptides and unique peptides in each protein and the corresponding protein sequence coverage are also indicated.

Compared to the results of the SDS-PAGE of PAXYB1 particles, 17 identified proteins corresponded to the protein bands in turn (Fig. 5, Fig. S3, and Table 3). These results convincingly demonstrated that these 17 proteins are related to particles of phage PAXYB1. These proteins, particularly the three proteins not previously identified, may reveal more details of the PAXYB1 structure.

## Discussion

The enormous variety of bacteriophages in environmental reservoirs provides us a successful phage-based antibacterial treatment strategy, which is dependent on the availability of new phages against different pathogen hosts. In this work, we isolated a new phage PAXYB1, which infects *P. aeruginosa*, and investigated its biological properties. PAXYB1 also has a strong ability to infect several clinical isolates of *P. aeruginosa*, which makes it promising for use in phage therapy. A structural analysis of PAXYB1 indicates that it is a new member of the *Podoviridae* family, exhibiting a morphological similarity to several other *P. aeruginosa* phages, such as MPK6, LUZ19 and phiKMV^27,29,30^.

The advancement of sequencing technology has allowed us to explore the genomic characterization of isolated phages more quickly and deeply, thus giving us better insights into their biological nature. Furthermore, a genomic analysis of phages may also provide us useful information in regard to their safety as potential therapeutics, such as excluding temperate phages or phages carrying toxins or antibiotic resistance genes^31^. PAXYB1 is likely lytic since no genes related to phage lysogeny were found in its genome. The PAXYB1 genome is intensely homologous with the genome of known members of phiKMV-like phages (Fig. 3, Fig. S2). PAXYB1’s genome information, together with its structural characteristics, lead to the conclusion that it is a new member of the phiKMV-like phage group^24^. The phiKMV-like viruses constitute an important genus of T7 related phages that infect *P. aeruginosa* and seem to be environmentally important as they appear ubiquitously and infect a wide range of *P. aeruginosa* strains^29^. Genomes of several phiKMV-like phages have been extensively analyzed, such as phiKMV, LKD16, LUZ19 and MPK6^18,22,26^.

To date, 15 members of phiKMV-like phages have been sequenced. PAXYB1 displays strong similarity at the nucleotide level (up to 90% identity) to other “phiKMV-like viruses” (Table S1). The early and early middle regions of PAXYB1 are different with other PhiKMV-like phages (Fig. 3, Fig. S2). All predicted genes’ functions remain unknown in the early cluster of PAXYB1. Only a few early middle proteins of known phiKMV-like phages have been expressed and examined in detail 21, 24, 28. Some early proteins of phages are gene regulators that interact with genes of the bacteria host. For example, T4 phage early proteins AsiA, MotA and Mrh can interact with sigma70 or sigma32 subunits of bacterial host RNA polymerase^32,33^. Early protein E3 of phage SPO1 led to growth inhibition when expressed in *E. coli* through targeting on host RNA polymerase^20,34^. Rybniker *et al*. identified three ORFs within an early operon of mycobacteriophage L5, which encodes gene products (gp77, gp78 and gp79) that are toxic to the host *Mycobacterium smegmatis* ^35^. The co-expression network between *P. aeruginosa* phage PaP3 and its host suggested that the early genes of PaP3 had the primary responsibility to inhibit the expression of host genes^36^. Later on, one early gene product of PaP3, gp70.1, was shown to have growth-inhibitory effects on *P. aeruginosa* and *E. coli*, which targets the host protein RpoS^37^. Phages’ early genes encoding growth inhibitors of bacteria may be used as antibacterial agents. The identification of their cellular targets might provide a tool for the rapid identification of promising drug targets in emerging pathogens, such as multidrug resistant *P. aeruginosa*. A survey revealed that most (64%) of the phage toxic proteins were encoded by phage early genes^38^. Therefore, we should further investigate the function of PAXYB1 early genes to determine the potential growth inhibitors of bacteria and toxic proteins in our future studies.

Phage PAXYB1 also encodes its own RNA polymerase (RNAP, ORF36), which is located at the end of the middle cluster that is a feature of the phiKMV-like phages. This phage may transcribe its late genes using its own RNAPs, similar to other phiKMV-like phages^27^. In the late region, PAXYB1 genes are organized in a very similar way with other known phiKMV-like phages that encode phage particle proteins and lysis proteins. Most of the predicted structural proteins of PAXYB1 were confirmed in the particle proteins by SDS-PAGE and mass spectrometry (Fig. 5, Table 3). We also found potential lysis cassette including endolysins, holin, Rz and Rz’ genes within late cluster of the PAXYB1 genome that is conserved amongst phiKMV-like viruses^39^. Interestingly, an analysis of one lysis protein gp57 (Rz protein) identified in PAXYB1 phage particles using mass spectrometry indicated that it may represent a component in PAXYB1 particles. Most of the known phiKMV-like phages have short or long gaps in late clusters compared to PAXYB1, particularly in genes encoding tail fiber proteins (gp50-52) (Table S2). Ceyssens’ study showed that some phiKMV-like phages are similar at the genome level, but they display a surprisingly large variation in various phenotypic properties, such as little overlap in host spectrum and different ability in escaping immune defence^20^. In particular, these phages had limited overlaps in the host spectrum even with almost identical tail fibers, implying that minor genomic changes can cause a significant shift in infectivity range^20^. Vitiello’s study also showed that only one amino acid substitution in a capsid protein of lambdaArgo phage strongly enhanced phage survival in the mouse circulatory system^40^. Differences in the tail fiber proteins of PAXYB1 with other phiKMV-like phages may lead to differences in killing susceptible strains or the ability to escape immune defence.

To date, the proteomic characterization of phiKMV-like viruses has not revealed any toxic gene products present in these bacteriophages including PAXYB1^21,24,28^. PhiKMV-like phages are good candidates in phage therapy. Numerous early studies demonstrated the therapeutic potential of phiKMV-like viruses in various animal infection models^18,20,29^, and no adverse side effects have yet been reported. For example, LUZ19 could infect clinical *P. aeruginosa* both *in vitro* and *in vivo* ^26^. In addition, Henry’s results showed that the bacteriophages isolated directly from the targeted host were the most efficient *in vivo*, supporting a personalized approach favouring an optimal treatment^26^. Merabishvili *et al*. evaluated the safety and efficacy of a bacteriophage cocktail (BFC-1, including PhiKMV-like phages) in the treatment of burn wound infections in a controlled clinical trial, and BFC-1 is active against the *P. aeruginosa* and *Staphylococcus aureus* strains in their model^41^. Alves’ studies suggest that the use of phage cocktails (including one phiKMV-like phage DL62) against *P. aeruginosa* could provide practical alternatives to antibiotic treatments for combating biofilm-related infections and in particular the devastating effects of biofilm-related CF infections^42^. Hence, further knowledge of the gene products of PhiKMV-like phages and their targets in bacteria are also extremely significant and helpful in antibacterial agent discovery and identifying potential toxic genes. Although the PAXYB1 phage has a very similar genome sequence with other phiKMV-like phages, it may have different characteristics, particularly with the three predicted particle proteins that were not previously identified and may reveal more details of the PAXYB1 structure and different interaction with host bacteria. Detecting the functions of unknown PAXYB1 genes and their interaction with the host strain are necessary before applying it to phage therapy.

## Materials and Methods

### Bacterial strains and culture conditions

The bacterial strains used in this study are listed in Table 1. Twenty clinical isolates of *P. aeruginosa* were isolated from clinical samples of patients in the Second Affiliated Hospital of Nanjing Medical University, Nanjing, China. All clinical strains were antibiotic resistant. Fourteen out of the 20 isolates were resistant to at least 2 of the anti-*pseudomonal* antibiotic classes tested, and the other 6 isolates were multidrug resistant. All strains were grown in Luria–Bertani (LB) medium at 37 °C.

### Isolation and propagation of bacteriophages

*P. aeruginosa* strain PAO1 was used as a host for phage isolation from wastewater in Nanjing. Sewage samples were filtered using 0.22 μm pore-size filters (Millipore, USA) to remove bacteria. One hundred microlitres of the filtrate was added to 5 ml of *P. aeruginosa* culture in early-log-phase at 37 °C for 24 h with constant shaking to enrich the phages. The culture was then centrifuged at 10,000 g for 20 min at 4 °C. Bacteriophages in the supernatant were tested for plaque formation using the double-layer agar plate method^43^. We picked a single isolated plaque on the double-layer plate to start the second (and subsequent) round of amplification. The infection cycle was repeated until the plaques were homogeneous (approximately 10 cycles). The phages were then amplified and stored at 4 °C.

### Host range analysis

The host range of PAXYB1 was tested against 20 clinical isolates of *P. aeruginosa* using the following method. Briefly, 100 μl of overnight bacterial cultures was mixed with 3 ml of molten top agar and overlaid on LB plates. Then, 10 μl of phage suspensions (approximately 10^10^ pfu/ml) was dropped onto the plates and allowed to air dry. Following overnight incubation at 37 °C, the plates were then examined for plaque formation to establish bacterial sensitivity to the phage. *P. aeruginosa* PAO1 and *E. coli* MG1655 were used as the positive and negative controls, respectively. All experiments were conducted according to the standard institutional guidelines of Nanjing Medical University (Nanjing, China). The study was approved by the research and ethics committee of the Second Affiliated Hospital of Nanjing Medical University, and informed consents were obtained from all the patients.

### Purification of phage PAXYB1

Purification of the phage PAXYB1 was performed as described previously with slight modifications^29^. Briefly, *P. aeruginosa* PAO1 culture at the early-log-phase (OD_600_ = 1.0) was infected by the PAXYB1 phage at 37 °C for 3 h with shaking. The cell debris was removed by centrifugation (14,000 g, 30 min, 4 °C). The supernatant passed through 0.22-μm-pore-size filters, yielding a crude extract of phage. Then, the phage crude extract was concentrated by ultracentrifugation, and the pellet containing the phage was suspended in SM buffer (5.8 g/L NaCl, 2 g/L MgSO_4_·7H_2_O, 50 ml/L 1 M pH7.5 TrisHCl). The concentrated suspension was further purified by cesium chloride gradient centrifugation. The phage zone was collected and ultracentrifuged to remove CsCl. Finally, the pellet was resuspended in SM buffer to yield the highly purified phage.

### Electron microscopy

The highly purified phage was spotted onto a carbon-coated copper grid and negatively stained with 2% (w/v) phosphotungstic acid. The micrographs were taken under FEI Tecnai G2 Spirit Bio TWIN transmission electron microscope at 80 kV.

### Thermostability

For testing PAXYB1 thermostability, the phage samples (approximately 10^10^ pfu/ml) were incubated at 37 °C, 45 °C, 50 °C, 55 °C, 60 °C and 65 °C for 30 min, respectively, and then tittered using the double-layer agar plate method to determine the phage survival rates.

### One-step growth curve

The one-step growth experiment was performed as described previously with slight modification^44^. In brief, *P. aeruginosa* PAO1 was grown in LB medium until the early-log-phase (1 × 10^8^ CFU/ml). Phage PAXYB1 was added to the PAO1 culture at a multiplicity of infection of 10 and allowed to adsorb for 10 min at 37 °C. Then, the mixture was centrifuged at 12,000 rpm for 1 min to remove un-adsorbed phages. After washing twice with fresh LB medium, the pellet of infected cells was resuspended in 50 ml of LB medium, and the culture was continuously incubated at 37 °C. Using the double-layer-agar plate method, we determined the free bacteriophage count at each time point. The latency period and burst period were obtained directly from these one-step growth curves. The burst size was calculated by dividing the phage titres at the plateau phase by the initial number of infective bacterial cells.

### Phage adsorption assay

The kinetics of phage PAXYB1 adsorption were assessed as described previously with slight modification^45^. The kinetics of phage PAXYB1 adsorption were determined by infecting *P.aeruginosa* PAO1 in LB broth at a multiplicity of infection of 0.01. Samples were taken at 1 min intervals, and titres were determined after the removal of cells by filtration of each sample through a 0.22 μm filter. The filtrates were diluted and spotted on a bacterial lawn of PAO1 to determine the titre of free phages in the culture.

### Bacteriophage *in vitro* lysis kinetics

*In vitro* lysis kinetics for PAXYB1were performed as described previously with slight modification^46^. Exponentially growing cultures of strain PAO1 were diluted with LB to an OD600 of 0.05. We added PAXYB1 phage suspension to obtain a multiplicity of infection of 0.001 and added an equal volume of LB medium to the control samples. The OD600 was recorded at 15 min intervals over a period of 6 hours.

### Extraction and sequencing of the PAXYB1 genome

The purified phage sample was treated with DNase I (New England Biolabs) and RNaseA (Tiangen Biotech) for 2 h at 37 °C to digest the exogenous DNA and RNA. The preparation was then treated with proteinase K (Tiangen Biotech) for 15 min at 55 °C. The phage genome DNA was further prepared with a TIANamp Bacteria DNA Kit (Tiangen Biotech). Restriction enzyme digestions of the genome DNA were performed according to the manufacturer’s instructions (Fermentas). DNA fragments were purified from agarose gels using a Gel Extraction Kit (Omega). The PAXYB1 genomic DNA was sequenced using an Illumina HiSeq. 2500 sequencer, and reads were assembled into a whole genome using SOAPdenovov2.04 software and GapCloserv1.12.

### PAXYB1 genome analysis

Putative open reading frames (ORFs) were predicted using artemis software (http://www.sanger.ac.uk/science/tools/artemis), with a threshold of 30 amino acids (aa) as a minimum for the length of protein. Function annotation was performed using the BLAST tools at NCBI (http://blast.ncbi.nlm.nih.gov/Blast.cgi) against the non-redundant protein sequences database. Transfer RNAs (tRNAs) were identified using tRNAscan-SE (v1.23, http://lowelab.ucsc.edu/tRNAscan-SE), and ribosome RNAs (rRNAs) were determined using RNAmmer (v1.2, http://www.cbs.dtu.dk/services/RNAmmer/). The ExPASy Compute pI/Mw tool was employed to calculate molecular masses and isoelectric points. Prokaryotic promoters were predicted using the BDGP prediction program^47^ (http://www.fruitfly.org/seq_tools/promoter.html). The whole viral nucleotide sequence similarities between phages were determined using megablast at NCBI. The global alignment of putative amino acid sequences was performed using the EMBOSS Needle tool at EMBL-EBI (European Molecular Biology Laboratory-European Bioinformatics Institute). Phylogenetic analyses between the genomes of related phages were performed with MEGA using the Neighbour-Joining algorithm.

### Structural protein analysis of PAXYB1

The highly purified phage sample was subjected to sodium dodecyl sulphate polyacrylamide gel electrophoresis (SDS-PAGE) using 12% acrylamide concentration. The gels were stained with silver as described by Shevchenko *et al*.^48^. For protein identification by liquid chromatography electrospray ionization with tandem mass spectrometry (LC-ESI MS/MS), the phage particles were digested with trypsin, and the trypticpeptides were analyzed by Q Exactive mass spectrometer (Thermo Scientific, USA). The corresponding ORFs were searched using the MASCOT engine (Matrix Science, London, UK; version 2.2) against the protein sequence library of PAXYB1.

### Nucleotide sequence accession number

The nucleotide sequence of the phage PAXYB1 genome has been deposited in GenBank under accession number KY618819.

## Electronic supplementary material

Supplementary Information
