# Supplementary material for: Characterization and genomic study of “phiKMV-Like” phage PAXYB1 infecting Pseudomonas aeruginosa
Source: Sci Rep. 2017 Oct 12;7:13068. doi: 10.1038/s41598-017-13363-7 (PMC5638911; doi:10.1038/s41598-017-13363-7)
Supplement: Supplementary file 1 — Supplementary Information [file 41598_2017_13363_MOESM1_ESM.pdf]

Characterization and genomic study of "phiKMV-Like" phage PAXYB1 infecting *Pseudomonas aeruginosa*

Xinyan Yu[1], Yue Xu[1], Yu Gu[1], Yefei Zhu[2], Xiaoqiu Liu[1]*

[1] Key Laboratory of Pathogen Biology of Jiangsu Province, Department of Microbiology, Nanjing Medical University, Nanjing 211166, People's Republic of China

[2] The Second Affiliated Hospital of Nanjing Medical University, Nanjing, China

Supplementary Fig. S1

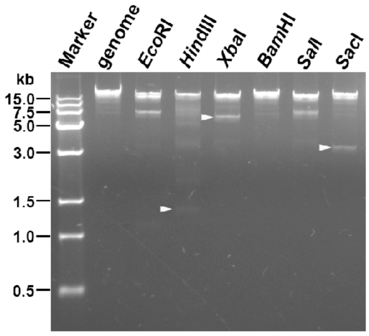

Fig. S1. Restriction digestion analysis of the PAXYB1 genome. The PAXYB1 genomic DNA was digested with *Eco*RI (2), *Hin*dIII (3), *Xba*I (4), *Bam*HI (5), *Sal*I (6) and *Sac*I (7), with the genome without digestion as a control (1). 15 kb DNA ladder marker was used as the standard (M).

Supplementary Fig. S2

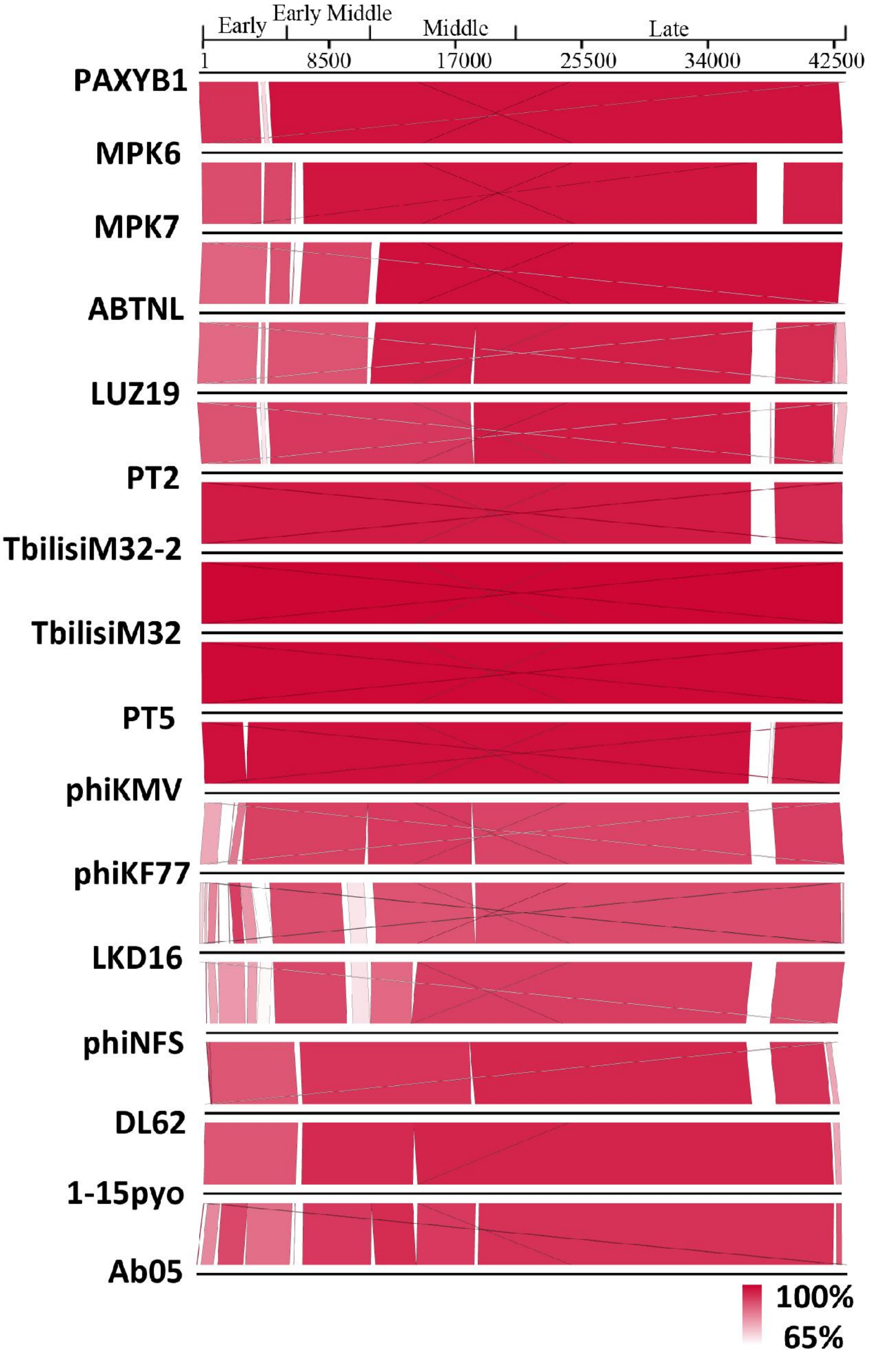

Supplementary Fig. S2. Whole-genome comparisons of all phiKMV-Like family phages using EasyFig. TbilisimM32-2 is Modified TbilisiM32.

Supplementary Fig. S3

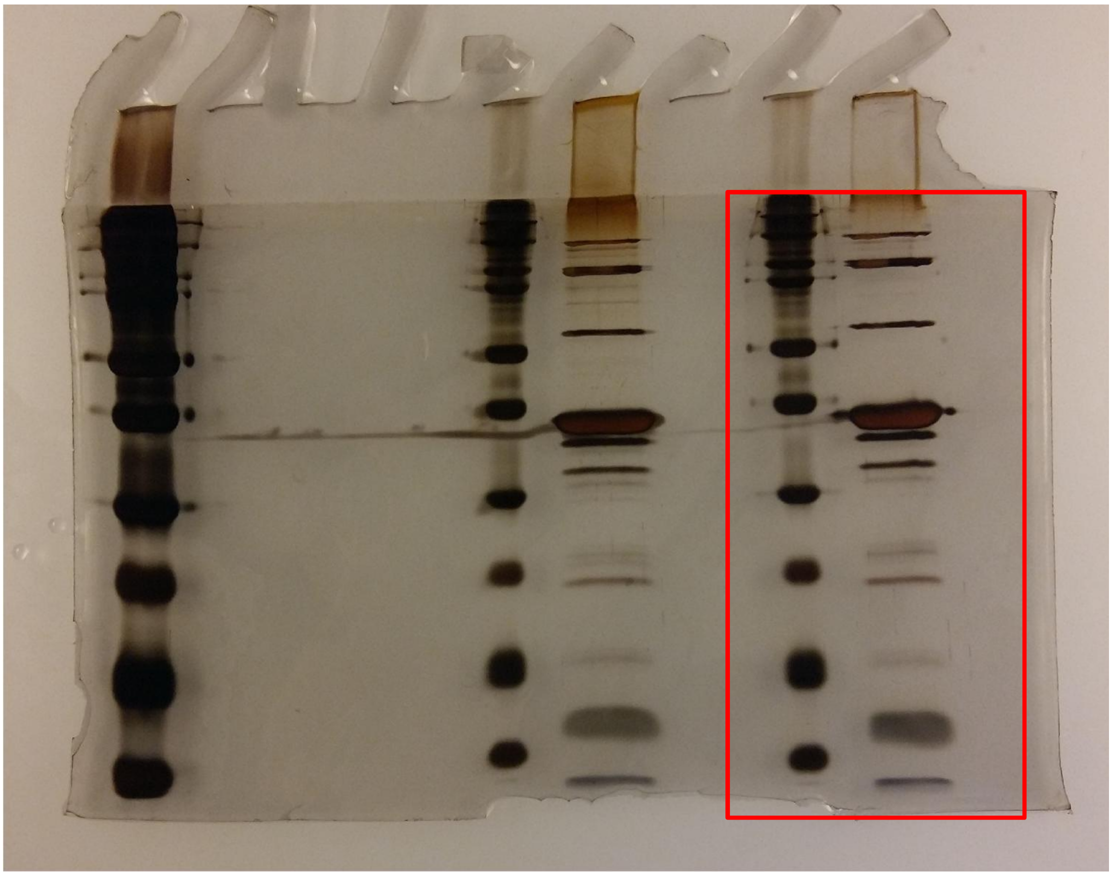

Supplementary Fig. S3. Original image for the silver staining of the purified PAXYB1 particles separated by SDS-PAGE (**Related to Fig. 5**). The area marked in red frame indicating the part shown in Fig.5.

Supplementary Table S1. Overview of φKMV-like phages included in this study.

Phage	Accession	Isolation site	Genome size (bp)	GC%	ORFs	tRNAs (n)	DTR (bp)	Query Coverage (%)	Identity (%)
PAXYB1	KY618819	Nanjing, China	43,337	62.3	60	0	468	100	100
ΦKMV	AJ505558	Moscow, Russia	42,519	62.3	48	0	414	93	94
LKD16	AM265638	Leuven, Belgium	43,200	62.6	54	0	428	86	91
LUZ19	AM910651	Leuven, Belgium	43,548	62.3	54	0	472	96	97
ΦKF77	FN263372	Moscow, Russia	43,152	62.9	53	0	452	96	92
PT2	EU236438	Tbilisi, Georgia	42,961	62.2	54	0	488	94	95
PT5	EU056923	Tbilisi, Georgia	42,954	62.3	52	0	413	97	94
ABTNL	KM067278	Dalian, China	43,227	62.4	54	0	453	92	98
Modified TbilisiM32	KX711710.1	Dalian, China	42965	62.3	54	0	ND	97	95
TbilisiM32	JQ307386	San Antonio, USA	42,966	62.3	51	0	423	97	94
Ab05	LN610574	Abidjan, Côte d'Ivoire	43,639	62.3	54	0	431	94	94
1-15pyo	LN610580	Orsay, France	42,750	62.2	49	0	ND	96	95
DL62	KR054031	Somerset, UK	42,508	62.2	55	0	ND	95	96
phiNFS	KU743887	Belfast, UK	42,351	62.3	49	0	ND	93	94
MPK6	JX997978	Seongnam-si, Korea	42,957	62.3	50	0	ND	97	98
MPK7	JX501340	Seongnam-si, Korea	42,874	62.1	53	0	ND	93	99

Supplementary Table S2. Comparison of genes encoding tail fiber proteins between PAXYB1 and other φKMV-like phages.

Phage	ORF49	ORF50	ORF51	ORF52
PAXYB1 49-52	100 (251aa)	100 (152aa)	100 (301aa)	100 (201aa)
MPK6 38-41	99.2 (251aa)	99.3 (152aa)	100 (301aa)	100 (201aa)
ΦKF77 38-41	100 (251aa)	98.7 (152aa)	98.7 (301aa)	100 (201aa)
LUZ19 38-41	100 (251aa)	98.7 (152aa)	98.3 (301aa)	100 (201aa)
LKD16 38-41	97.6 (251aa)	56.6 (152aa)	73.5 (302aa)	88.1 (201aa)
TbilisiM32 38-41	94.4 (251aa)	50.0 (152aa)	71.2 (302aa)	98.0 (201aa)
DL62 59.1-62.1	99.6 (251aa)	50.0 (152aa)	70.9 (302aa)	88.6 (201aa)
Ab05 43-46	99.6 (251aa)	50.0 (152aa)	70.5 (302aa)	88.6 (201aa)
PT5 38-41	94.4 (251aa)	50.0 (152aa)	66.6 (281aa)	97.5 (201aa)
1-15pyo 39-41	83.7 (225aa)	46.6 (163aa)	71.2 (302aa)	ND
MPK7 43-46	96.8 (251aa)	30.0 (146aa)	27.3 (292aa)	47.3 (196aa)
ABTNL 44-47	96.8 (251aa)	28.8 (146aa)	26.3 (292aa)	47.3 (196aa)
phiNFS 39-42	95.2 (251aa)	28.1 (146aa)	24.9 (291aa)	50.2 (196aa)
PT2 38-41	95.2 (251aa)	28.1 (146aa)	25.3 (292aa)	34.3 (130aa)
ΦKMV 38-41	95.2 (251aa)	28.1 (146aa)	24.9 (292aa)	34.3 (139aa)

The homologues of the four assumed tail fiber proteins of PAXYB1 and other phages are searched using BLASTp analysis. The homologues of gp52 in MPK7, ABTNL, phiKF77, PT2 and KMV are not found using BLASTp, and obtained throughout analysis of the genomes. The global identity values between amino acid sequences are determined by EMBOSS Needle tool at EMBL-EBI.
